# Associations of Home-Based Individual and Family Exercise with Personal and Family Well-Being Amid the COVID-19 Pandemic: A Population-Based Survey in Hong Kong

**DOI:** 10.3390/bs15030376

**Published:** 2025-03-17

**Authors:** Jinzhou Yu, Katherine Yuk-Ping Sze, Agnes Yuen-Kwan Lai, Shirley Man-Man Sit, Wei-Jie Gong, Jia Guo, Tai-Hing Lam, Man-Ping Wang, Sai-Yin Ho

**Affiliations:** 1 School of Nursing, The University of Hong Kong, Hong Kong, China; yjz1997@connect.hku.hk (J.Y.); ypsze215@hku.hk (K.Y.-P.S.); 2 School of Nursing and Health Sciences, Hong Kong Metropolitan University, Hong Kong, China; ayklai@hkmu.edu.hk; 3 School of Public Health, The University of Hong Kong, Hong Kong, China; ssit@connect.hku.hk (S.M.-M.S.); hrmrlth@hku.hk (T.-H.L.); syho@hku.hk (S.-Y.H.); 4 Department of Family Medicine, Medical School, Shenzhen University, Shenzhen 518060, China; gweijie@connect.hku.hk; 5 Xiangya School of Nursing, Central South University, Changsha 410013, China; guojia621@163.com

**Keywords:** exercise, family well-being, personal happiness, adversity coping capability, psychological distress

## Abstract

Exercise benefits individuals, but research about exercising with family members (EFM) and personal and family well-being is scarce. We investigated the associations of home-based individual exercise (IE) and EFM with personal and family well-being amid the COVID-19 pandemic. A population-based cross-sectional survey on Hong Kong Chinese adults was conducted from February to March 2021, when COVID-19 was under control (N = 5825). Respondents reported the number of days IE and EFM occurred at home in the past 7 days (0, 1–2, 3–4, 5–6, 7), respectively. Family well-being, personal happiness, and personal/family adversity coping capability (ACC/FACC) were each measured with an 11-point scale (range 0–10). Psychological distress was measured using the four-item Patient Health Questionnaire (range 0–12). Associations were assessed using multiple linear regressions. Of 1911 respondents, 9.2% reported having ≥3 days of EFM. After adjusting for each other and sociodemographic characteristics, more frequent IE or EFM (≥3 vs. 0–2 days) was associated with greater family well-being, FACC, personal happiness, and lower psychological distress. Our findings suggest that home-based EFM or IE may promote personal/family well-being and highlight the potential for incorporating home-based exercise into public health strategies to enhance overall well-being.

## 1. Introduction

Mental health is a state of well-being that allows individuals to handle life’s stresses, realize their potential, work and learn effectively, and contribute to their community (World Health Organization, 2022a). Personal happiness is part of the subjective well-being construct, which is related to the quality of one’s life (Abdel-Khalek, 2006). Family well-being is conceptualized as family functioning or family quality of life, which includes family health, family happiness, and family harmony (Hoffman et al., 2006; Lam et al., 2012). The family is crucial in determining an individual’s emotional and psychological well-being (Thomas et al., 2017). The COVID-19 pandemic threatens the perceived mental health and well-being of individuals and families worldwide (Prime et al., 2020; Vindegaard & Benros, 2020). According to our previous surveys—Family amidst COVID-19 survey (FamCov-1) and the Hong Kong Family and Health Information Trend Survey (FHInTS)—the mental health burdens of families and individuals mounted on social unrest and economic insecurity during the pandemic, with a surge in the prevalence of severe psychological distress, a state of emotional suffering typically characterized by symptoms of depression, anxiety, and stress (Drapeau et al., 2012), in Hong Kong (Wong et al., 2021; Zhao et al., 2020). One-third of respondents reported an increase in negative family emotions, nearly one-fifth reported a decline in family well-being, and those reporting personal unhappiness appeared to have increased compared with 2016 and 2017 (Wong et al., 2021; Zhao et al., 2020).

Exercise is pivotal to preventing and managing mental illnesses (World Health Organization, 2022b). During the COVID-19 pandemic, exercise was found to be negatively associated with depression and anxiety symptoms, and positively associated with happiness, life satisfaction, and resilience in individuals (Lancaster & Callaghan, 2022; Mutz, 2021; Rösel et al., 2022). Globally, the COVID-19 pandemic negatively impacted physical activity (PA) levels (Vancini et al., 2021), and Hong Kong was no exception. Studies in Hong Kong showed that after the outbreak, compared to 2019, the frequency of walking and high-intensity exercise decreased among the general population, with similar declines observed in the elderly and children (Lee et al., 2021; J. Wang et al., 2021). On the one hand, stay-at-home orders, social distancing, and the closure of parks and gyms effectively reduced the spread of infection but also limited outdoor activities, disrupting exercise routines. On the other hand, these measures increased interest in home-based exercise. Home-based exercise refers to exercise or physical activities conducted within or near the household, including the garden and/or driveway (Denton et al., 2021; Pu et al., 2020), and it was seen as an important way to maintain physical and mental health with fewer environmental constraints during the pandemic (Carvalho & Gois, 2020). Results of meta-analyses show that home-based exercise is feasible, sustainable, and low-cost (de Almeida et al., 2020; Feng et al., 2019).

This shift highlighted the importance of exploring the potential benefits of home-based exercise on the well-being of family members and the overall family. The family has a strong influence on the exercise behaviors of its members (van Sluijs et al., 2011) and family engagement results in higher level exercise and sustained behavior change (Gardner et al., 2019; Rhodes & Lim, 2018). However, the relationship between perceived well-being and home-based exercise, particularly when exercising with family members (EFM), remains unclear. This research gap about home-based EFM warrants further investigation, especially as people spent more time with their families due to COVID-19-related social distancing and closure of public facilities (Hong Kong Special Administrative Region, 2021; Leung et al., 2022; Zhang et al., 2021). Understanding this relationship could provide valuable insights for developing effective home-based exercise interventions aimed at improving both mental health and family well-being and offering an alternative strategy for public health promotion that can be utilized both during and beyond future pandemic conditions.

Therefore, the present study aimed to explore the associations of home-based individual exercise (IE) and EFM with family well-being, family adversity coping capability (FACC), personal happiness, adversity coping capability (ACC), and psychological distress. ACC/FACC refers to an individual’s or family’s perceived capability to cope with adversity or stressful events, which is related to personal and family well-being (Gong et al., 2022; Walsh, 1996).

## 2. Materials and Methods

### 2.1. Study Design and Procedures

We conducted the population-based Family amid COVID-19 Survey 2 (FamCov-2) under the Jockey Club SMART Family-Link project from 22 February to 23 March 2021. Details of this survey have been reported elsewhere (Chen et al., 2023; Gong et al., 2022; Leung et al., 2022). Briefly, the Hong Kong Public Opinion Research Institute (HKPORI), a well-known local survey agency, used landlines, mobile phones, and online methods to survey Hong Kong residents aged 18 years or older who can read or communicate in Chinese. FamCov-2 aimed to investigate the individual and family well-being and related factors and behaviors of Hong Kong residents during the 4 weeks when the fourth wave of the COVID-19 pandemic was under control in Hong Kong. Of 2420 valid phone numbers, 1522 participants (62.9%) completed the telephone interview. Of 48,825 email invitations opened, 6013 (12.3%) respondents completed the entire survey. The questionnaire has a core section and three subsets: family communication, COVID-19 information, and COVID-19 influence. Each respondent was randomly assigned to answer one subset of questions in addition to the core questions. While individual exercise was a core item (5825 respondents), family exercise was an item under the family communication subset (1911 respondents). Ethics approval was obtained from the Institutional Review Board of The University of Hong Kong/Hospital Authority Hong Kong West Cluster (Reference Number: UW 20-651), and all respondents gave informed consent before starting the survey. All methods were performed in accordance with the relevant guidelines and regulations.

### 2.2. Measurements

IE was measured using the question “In the past 7 days, how many days did you do exercise at home? (Simple stretching, movements, or active games are also considered exercise.)”. EFM was measured likewise: “In the past 7 days, how many days did you do exercise with your family members at home? (Simple stretching, movements, or active games are also considered exercise)”. Response options included: 0 day, 1–2 days, 3–4 days, 5–6 days, and 7 days^1^. During the COVID-19 period, according to the recommended moderate exercise volume per week (Hull et al., 2020), the frequency of exercise is categorized as low frequency (0–2 days), moderate frequency (3–4 days), and high frequency (≥5 days) for analysis purposes.

Personal happiness was measured using a single item with proven reliability and validity (Abdel-Khalek, 2006): “How happy do you think you are?”. Family well-being was measured using the mean score of three separate questions that asked “How healthy/harmonious/happy do you think your family is? (3Hs)”, which were reliable (Cronbach’s α coefficient = 0.89) and valid in previous studies (Lam et al., 2012; X. Wang et al., 2016). The Cronbach’s α coefficient in the present study was 0.94. ACC is an individual’s perceived ability to cope with adversity or stressful events (Gong et al., 2022), while FACC reflects the family’s ability to handle such situations. ACC and FACC were measured using a single item “How do you rate your/your family’s capability to cope with adversities?”, respectively. ACC was found to be valid in the previous study (Gong et al., 2022). All of the above outcomes were assessed using 11-point scales (ranging from 0 to 10), with higher scores indicating better results.

The Chinese version of the 4-item Patient Health Questionnaire (PHQ-4) was used to assess psychological distress, which includes two 2-item subscales that measure anxiety and depressive symptoms and has shown good reliability and validity (Luo et al., 2019; Yu et al., 2011). Participants were asked to rate the frequency of the mentioned symptoms in the past 2 weeks using a 4-point Likert scale, where 0 represents “not at all” and 3 represents “nearly every day”. The total PHQ-4 score ranged from 0 to 12, with higher scores indicating greater psychological distress. In the present study, PHQ-4 had a Cronbach’s α coefficient of 0.88, while the anxiety and depressive symptoms subscales had coefficients of 0.82 and 0.79.

Sociodemographic characteristics included sex; age; educational attainment (primary or lower, secondary, diploma or certificate, associate degree, and degree or higher), which was recoded into secondary or below and tertiary for analysis; employment status; monthly household income per person, which was dichotomized as lower and higher using the size-specific median monthly household income from the 2019 Hong Kong census data (median monthly household income = HKD 20,000, USD 1  =  HKD 7.8) (Census and Statistics Department, 2019a); housing type (rented/owned); and the number of cohabitants (excluding the respondent).

### 2.3. Statistical Analysis

All data were weighted to the distribution of sex, age, and education in the 2019 Hong Kong population census (Census and Statistics Department, 2019b, 2019c). The distribution of sociodemographic characteristics, individual/family exercise frequency, and outcome variables was presented through descriptive analysis, showing percentages for categorical variables and mean ± standard deviation (SD) for continuous variables. Pairwise comparisons using Chi-square tests were used to compare the sociodemographic characteristics and the frequency of having IE and EFM. ANOVAs and t-tests were used to compare mean scores on family well-being, FACC, personal happiness, ACC, and psychological distress.

Multiple linear regressions (MLR) were used to assess the associations of IE and EFM with personal and family well-being variables. The results were reported as unstandardized regression coefficients (Bs) and their 95% confidence intervals (CIs). MLR is a statistical method that models the relationship between one continuous dependent variable and two or more independent variables. By including multiple variables, MLR can account for the influence of other variables, isolating the effect of each independent variable (Cohen et al., 2013).

To fully investigate the effects of EFM and IE on personal and family well-being, two MLR models were built, in which IE/EFM was coded into two ordinal variables: the first with three categories (low frequency: 0–2 days, moderate frequency: 3–4 days, and high frequency: ≥5 days) and the second with two categories (low frequency: 0–2 days and moderate to high frequency: ≥3 days), with low frequency being set as the reference group. In Model 1, either IE or EFM was treated as an independent variable, and personal and family well-being outcomes were treated as dependent variables, with sociodemographic characteristics included as covariates. Model 2 set both IE and EFM as independent variables to eliminate the confounding effects they might have on each other, with the same dependent variables and covariates as in Model 1. All statistical analyses were conducted using Stata MP 17.0 (StataCorp LP, College Station, TC, USA), with a two-tailed P < 0.05 indicating statistical significance.

## 3. Results

### 3.1. Respondents’ Characteristics by the Frequency of Home-Based Exercise

Table 1 shows that of 5825 respondents, after weighting, 51.9% were female, 81.1% were aged 18–64 years, 61.2% had secondary or primary education, 57.1% were employed, 56.8% had a lower monthly household income, 57.9% lived in owned housing, and 77.9% had 1–3 cohabitants. Of all respondents, 54.8% reported low (0–2 days), 18.2% moderate (3–4 days), and 27.0% high (≥5 days) frequency of IE in the past 7 days. Of 1911 respondents with data on EFM, 90.7% reported low frequency, 4.9% moderate, and 4.3% high. More respondents with high versus low frequency IE were female (28.5 vs. 25.4%), at older age, retired, had low education level (32.6 vs. 18.1%), lower monthly household income (30.3 vs. 22.6%), owned housing (28.1 vs. 25.5%), and had fewer cohabitants (all Ps ≤ 0.01). More respondents with high versus low frequency EFM were male (6.5 vs. 2.1%), at older age, retired, had low education level (5.5 vs. 2.5%), lower monthly household income (5.7 vs. 2.4%), and had more cohabitants (all Ps < 0.05).

### 3.2. Comparison of Personal and Family Well-Being by Respondents’ Characteristics

Table 2 shows that females reported higher happiness (mean ± SD, 5.89 ± 1.93 vs. 5.55 ± 2.17) but lower ACC (6.12 ± 1.79 vs. 6.30 ± 1.89) (Ps < 0.001). The lower education group had higher family well-being (6.65 ± 1.95 vs. 6.44 ± 1.91) and lower psychological distress (2.70 ± 2.99 vs. 3.40 ± 2.79), but lower ACC (6.01 ± 1.96 vs. 6.28 ± 1.80) (Ps < 0.001). Those of older age, higher monthly household income, and who owned a house reported greater family well-being, FACC, personal happiness, ACC, and lower psychological distress (Ps < 0.01).

### 3.3. The Associations of the Frequency of Home-Based Exercise with Personal and Family Well-Being

Table 3 shows the associations of IE or EFM with family well-being, FACC, personal happiness, ACC, and psychological distress. After adjusting for sociodemographic characteristics (Model 1), moderate to high (≥3 vs. 0–2 days) frequency IE or EFM (versus low) were significantly associated with greater family well-being (B_IE_ = 0.46, 95% CI [0.29, 0.63]; B_EFM_ = 0.96, [0.66, 1.27]; Ps < 0.001), FACC (B_IE_ = 0.43, [0.26, 0.60]; B_EFM_ = 0.77, [0.47, 1.08]; Ps < 0.001), personal happiness (B_IE_ = 0.29, [0.10, 0.48]; B_EFM_ = 0.54, [0.20, 0.88]; Ps < 0.01), ACC (B_IE_ = 0.41, [0.24, 0.58]; B_EFM_ = 0.48, [0.18, 0.77]; Ps < 0.01), and lower psychological distress (B_IE_ = −0.45, [−0.70, −0.19]; B_EFM_ = −0.80, [−1.25, −0.34]; Ps < 0.01) (all P for trend < 0.01). With further mutual adjustment between IE and EFM (Model 2), the associations of more frequent (≥3 vs. 0–2 days) IE or EFM with greater family well-being (B_IE_ = 0.32, [0.14, 0.50]; B_EFM_ = 0.79, [0.46, 1.11]; Ps < 0.01), FACC (B_IE_ = 0.33, [0.15, 0.51]; B_EFM_ = 0.59, [0.27, 0.91]; Ps < 0.001), personal happiness (B_IE_ = 0.22, [0.02, 0.42]; B_EFM_ = 0.42, [0.06, 0.77]; Ps < 0.05), ACC (B_IE_ = 0.36, [0.18, 0.54]; P < 0.001), and lower psychological distress (B_IE_ = −0.34, [−0.61, −0.07]; B_EFM_ = −0.61, [−1.09, −0.13]; Ps < 0.05) remained, but the association of EFM with ACC became statistically non-significant.

## 4. Discussion

We have first shown that home-based EFM and IE were independently associated with greater family well-being, FACC, personal happiness, and lower psychological distress. Our study also showed that IE was associated with greater ACC. After mutual adjustment, no association between EFM and ACC was found.

In Model 2, the moderate to high frequency EFM group showed a 0.42 increase in personal happiness and a 0.61 decrease in psychological distress, while the IE group showed a 0.22 increase and a 0.34 decrease, respectively. Recent studies indicated that home-based exercise was associated with less anxiety, depression, and stress caused by social isolation and greater self-efficacy and subjective happiness (Cao et al., 2023; Kikuchi et al., 2023). During the COVID-19 pandemic, those who engaged in moderate exercise with others or alone had higher continuous mental health scores than those who were inactive (Lesser & Nienhuis, 2020). Compared to the low frequency exercise group in Model 2, the moderate to high frequency EFM group saw increases in family well-being and FACC by 0.79 and 0.59, respectively, while the corresponding IE group saw increases of 0.32 and 0.33, respectively. A previous study during the COVID-19 pandemic has proven that exercise between parents and children was associated with greater family relationships and parents’ happiness and less loneliness (Koga et al., 2023). Exercise is considered a means of developing social skills (Opstoel et al., 2020) and exercising with family members was associated with greater family communication and perceived health (Lai et al., 2020). In addition, positive emotions, such as happiness, are contagious within the family context, and family members are more likely to transmit happiness rather than pain (Chi et al., 2019). However, we noticed that the absolute B values of EFM in Model 2 decreased from moderate to high frequency in personal happiness and psychological distress. This may be because personal and family well-being are multifaceted constructs shaped by dynamic interactions between personal and social factors (Musek & Polic, 2014; Wollny et al., 2010), and exercise is just one influencing factor.

We also found that more frequent home-based IE was associated with ACC, which was consistent with previous research findings (Perchtold-Stefan et al., 2020). Regularly engaging in exercise was associated with better emotional regulation and adaptive stress coping ability through cognitive reappraisal (Perchtold-Stefan et al., 2020). The present study found no association between EFM and ACC after adjusting for IE, and further studies are needed to confirm this result.

We found that more males reported high frequency EFM, while more females reported high frequency IE. In addition, more older adults (≥65) reported high frequency EFM, which was consistent with previous findings about IE (Smith et al., 2020; Wijngaards et al., 2022). Home-based exercise provided an alternative and effective way for older people to maintain necessary physical activity during the pandemic (Ghram et al., 2021), and should also be considered as one of the ways to improve the health of older people in the post-COVID-19 era. More respondents with low education level and monthly household income reported high frequency EFM, which was different from another finding on IE during the pandemic (Wijngaards et al., 2022).

Exercise is one of several contributing factors to personal and family well-being, and home-based exercise might be an alternative for those who cannot go to the gym or have limited time for exercising, as it is more convenient, cost-effective, and sustainable (Feng et al., 2019; Santos et al., 2023). Home-based exercise has been used as an intervention to promote family members’ mental health with positive results and appreciation from participants (Madruga et al., 2021; Moltrecht et al., 2024). However, EFM may not be equally beneficial for all groups, since different gender, cultural, or social norms may impact the experiences during the process (Petty & Trussell, 2021). Current research on home-based exercise is limited to the elderly, children, and those with mobility impairments, and the research quality is constrained due to insufficient sample size and effect size (Moltrecht et al., 2024). Further research is needed to verify the benefits of home-based exercise with family members, and the development and testing of interventions using family exercise to promote personal and family well-being in the post-COVID-19 pandemic era are warranted.

Our study had several limitations. First, the frequency of home-based IE and EFM was self-reported, which makes it susceptible to social desirability, recall bias, and measurement bias. Second, the intensity and duration of exercise were not measured. Given the typical multi-storey small unit housing in Hong Kong, exercises were likely limited to simple movements, resistance training using small equipment (e.g., dumbbells) or body weight (e.g., sit up, push up), and stretching, etc. For future studies, combining objective measurement tools (e.g., heart rate monitors) with standardized self-report questionnaires (e.g., the International Physical Activity Questionnaire, IPAQ) is suggested to enhance data accuracy and reliability. Third, EFM was measured only in one subset of the questionnaire, with a smaller sample size. Fourth, causal relations cannot be inferred due to the cross-sectional study design, and well-designed randomized controlled trials (RCTs) are needed for future research. Fifth, the study sample consisted of Chinese individuals from Hong Kong, and the data were collected during the COVID-19 pandemic; whether our results could be generalized to other settings is uncertain.

## 5. Conclusions

Our results on the associations of home-based EFM or IE with family well-being, FACC, personal happiness, ACC, and psychological distress suggest that home-based EFM or IE may contribute to personal and family well-being, but the reverse association is also plausible. Prospective studies are warranted to clarify temporality. The development and testing of interventions using home-based EFM or IE to promote personal and family well-being are warranted. Our findings also highlight the potential for incorporating home-based exercise into public health strategies to enhance overall well-being.

## Figures and Tables

**Table 1:** Weighted sociodemographic characteristics’ distribution by individual exercise and exercising with family members ^1^.

	IE (Weighted N = 5474)						EFM (Weighted N = 1800)					
	Low Frequency (3001, 54.8%)	Moderate Frequency (994, 18.2%)		High Frequency (1479, 27.0%)		Total	Low Frequency (1633, 90.7%)	Moderate Frequency (89, 4.9%)		High Frequency (78, 4.3%)		Total
	n (%)	n (%)	P ^2^ (vs. Low Frequency)	n (%)	P (vs. Low Frequency)	n (%)	n (%)	n (%)	P ^2^ (vs. Low Frequency)	n (%)	P (vs. Low Frequency)	n (%)
Sex			0.04		0.003				0.35		<0.001	
Male	1504 (57.1)	460 (17.5)		670 (25.4)		2634 (48.1)	798 (88.2)	48 (5.3)		59 (6.5)		905 (50.2)
Female	1498 (52.7)	534 (18.8)		808 (28.5)		2840 (51.9)	836 (93.3)	41 (4.6)		19 (2.1)		896 (49.8)
Age (years)			<0.001		<0.001				0.013		<0.001	
18–24	320 (70.6)	82 (18.1)		51 (11.3)		453 (8.3)	135 (97.1)	2 (1.4)		2 (1.4)		139 (7.7)
25–34	598 (71.2)	137 (16.3)		105 (12.5)		840 (15.3)	277 (94.5)	7 (2.4)		9 (3.1)		293 (16.3)
35–44	662 (66.0)	198 (19.7)		143 (14.3)		1003 (18.3)	308 (91.1)	23 (6.8)		7 (2.1)		338 (18.8)
45–54	623 (61.3)	179 (17.6)		214 (21.1)		1016 (18.6)	300 (91.5)	15 (4.6)		13 (4.0)		328 (18.2)
55–64	522 (46.2)	242 (21.4)		367 (32.4)		1131 (20.7)	343 (89.8)	19 (5.0)		20 (5.2)		382 (21.2)
≥65	276 (26.7)	158 (15.3)		598 (57.9)		1032 (18.8)	272 (84.5)	23 (7.1)		27 (8.4)		322 (17.9)
Educational attainment			0.02		<0.001				0.27		0.002	
Secondary/below	1665 (49.7)	594 (17.7)		1093 (32.6)		3352 (61.2)	987 (89.2)	59 (5.3)		61 (5.5)		1107 (61.5)
Tertiary	1337 (63.0)	401 (18.9)		385 (18.1)		2123 (38.8)	646 (93.2)	30 (4.3)		17 (2.5)		693 (38.5)
Employment status			<0.001		<0.001				0.046		0.005	
In paid employment	2010 (64.4)	542 (17.4)		571 (18.3)		3123 (57.1)	958 (92.6)	44 (4.3)		33 (3.2)		1035 (57.5)
Full-time students	213 (71.2)	51 (17.1)		35 (11.7)		299 (5.5)	88 (95.7)	2 (2.2)		2 (2.2)		92 (5.1)
Housekeeper	224 (41.6)	108 (20.0)		207 (38.4)		539 (9.8)	144 (87.3)	13 (7.9)		8 (4.8)		165 (9.2)
Retired	367 (30.8)	232 (19.5)		592 (49.7)		1191 (21.8)	334 (85.9)	26 (6.7)		29 (7.5)		389 (21.6)
Unemployed	187 (58.3)	60 (18.7)		74 (23.1)		321 (5.9)	110 (91.7)	4 (3.3)		6 (5.0)		120 (6.7)
Monthly household income			0.55		<0.001				0.85		<0.001	
Lower (<median)	1619 (52.1)	547 (17.6)		943 (30.3)		2305 (42.1)	935 (89.5)	50 (4.8)		60 (5.7)		1045 (58.0)
Higher (≥median)	1383 (58.5)	447 (18.9)		535 (22.6)		3170 (57.9)	699 (92.5)	39 (5.2)		18 (2.4)		756 (42.0)
Housing type			0.10		0.01				0.48		0.15	
Rent	1312 (56.9)	405 (17.6)		588 (25.5)		2192 (37.6)	723 (90.4)	36 (4.5)		41 (5.1)		800 (44.4)
Own	1690 (53.3)	589 (18.6)		891 (28.1)		3633 (62.4)	910 (91)	53 (5.3)		37 (3.7)		1000 (55.6)
Number of cohabitants			0.18		<0.001				0.33		0.028	
0	204 (53.4)	62 (16.2)		116 (30.4)		382 (7.0)	116 (97.5)	3 (2.5)		0 (0.0)		119 (6.6)
1–3	2295 (53.8)	788 (18.5)		1180 (27.7)		4263 (77.9)	1273 (90.6)	71 (5.1)		61 (4.3)		1405 (78.1)
≥4	502 (60.6)	144 (17.4)		182 (22.0)		828 (15.1)	244 (88.4)	16 (5.8)		16 (5.8)		276 (15.3)

FACC—family adversity coping capability; ACC—adversity coping capability; IE—individual exercise; EFM—exercising with family members. ^1^ Missing data were excluded. Percentages may not total 1 after rounding. Frequency may not add up to the total numbers after weighting. Data were weighted by the sex, age, and education distribution of the 2019 Hong Kong general population. ^2^ Chi-square test.

**Table 2:** Comparison of family well-being, family adversity coping capability, personal happiness, adversity coping capability, and psychological distress by sociodemographic characteristics ^1^.

	Family Well-Being (0–10)		FACC (0–10)		Personal Happiness (0–10)		ACC (0–10)		Psychological Distress (0–12)	
	Mean ± SD	P ^2^	Mean ± SD	P ^2^	Mean ± SD	P ^2^	Mean ± SD	P ^2^	Mean ± SD	P ^2^
Sex		0.94		0.22		<0.001		<0.001		0.10
Male	6.49 ± 1.90		6.38 ± 1.92		5.55 ± 2.17		6.30 ± 1.89		3.16 ± 2.91	
Female	6.50 ± 1.94		6.44 ± 1.92		5.89 ± 1.93		6.12 ± 1.79		3.29 ± 2.80	
Age (years)		<0.001		<0.001		<0.001		<0.001		<0.001
18–24	5.87 ± 2.02		5.79 ± 2.01		5.39 ± 1.90		5.67 ± 1.79		4.42 ± 2.86	
25–34	6.00 ± 2.02		5.97 ± 2.09		5.38 ± 2.05		5.99 ± 1.80		4.12 ± 2.82	
35–44	6.33 ± 1.92		6.27 ± 1.93		5.41 ± 2.11		6.09 ± 1.85		3.70 ± 2.86	
45–54	6.62 ± 1.83		6.49 ± 1.78		5.70 ± 2.07		6.32 ± 1.80		3.02 ± 2.77	
55–64	6.91 ± 1.70		6.84 ± 1.66		6.15 ± 1.93		6.54 ± 1.77		2.30 ± 2.52	
≥65	7.17 ± 1.78		7.03 ± 1.85		6.46 ± 1.95		6.43 ± 1.97		1.84 ± 2.52	
P for trend		<0.001		<0.001		<0.001		<0.001		<0.001
Educational attainment		<0.001		0.16		0.14		<0.001		<0.001
Secondary/below	6.65 ± 1.95		6.47 ± 1.95		5.79 ± 2.08		6.01 ± 1.96		2.70 ± 2.99	
Tertiary	6.44 ± 1.91		6.39 ± 1.91		5.70 ± 2.05		6.28 ± 1.80		3.40 ± 2.79	
Employment status		<0.001		<0.001		<0.001		<0.001		<0.001
In paid employment	6.38 ± 1.91		6.32 ± 1.91		5.62 ± 2.04		6.23 ± 1.80		3.41 ± 2.81	
Full-time students	5.80 ± 1.98		5.80 ± 2.02		5.37 ± 1.91		5.68 ± 1.81		4.41 ± 2.79	
Housekeeper	7.17 ± 1.81		6.83 ± 1.86		6.15 ± 1.92		6.12 ± 1.94		2.67 ± 2.82	
Retired	7.12 ± 1.74		7.00 ± 1.75		6.38 ± 1.98		6.52 ± 1.86		1.95 ± 2.46	
Unemployed	6.00 ± 2.03		5.93 ± 2.04		4.82 ± 2.15		5.61 ± 1.94		4.22 ± 3.22	
Monthly Household income		<0.001		<0.001		<0.001		<0.001		0.008
Lower (<median)	6.25 ± 2.04		6.14 ± 2.02		5.55 ± 2.13		5.93 ± 1.88		3.34 ± 3.05	
Higher (≥median)	6.67 ± 1.81		6.61 ± 1.82		5.85 ± 2.00		6.41 ± 1.79		3.14 ± 2.70	
Housing type		<0.001		<0.001		<0.001		<0.001		<0.001
Rent	6.25 ± 2.00		6.10 ± 2.05		5.55 ± 2.07		6.04 ± 1.84		3.51 ± 2.94	
Own	6.64 ± 1.86		6.60 ± 1.82		5.83 ± 2.05		6.31 ± 1.83		3.05 ± 2.79	
Number of cohabitants		<0.001		<0.001		0.13		0.40		0.71
0	5.92 ± 2.04		6.03 ± 2.08		5.54 ± 2.14		6.11 ± 1.96		3.30 ± 3.02	
1–3	6.55 ± 1.89		6.46 ± 1.89		5.73 ± 2.04		6.21 ± 1.83		3.23 ± 2.83	
≥4	6.43 ± 1.99		6.35 ± 2.02		5.79 ± 2.15		6.26 ± 1.84		3.16 ± 2.95	
P for trend		0.005		0.12		0.07		0.19		0.41

FACC—family adversity coping capability; ACC—adversity coping capability; SD—standard deviation. ^1^ Missing data were excluded. Percentages may not total 1 after rounding. ^2^ Independent sample t-tests and one-way ANOVA were performed on unweighted samples.

**Table 3:** Associations of the frequency of home-based exercise with family well-being, family adversity coping capability, personal happiness, adversity coping capability, and psychological distress ^1^.

	Family Well-Being (0–10)					FACC (0–10)			
	Model 1 ^2^			Model 2 ^3^		Model 1		Model 2	
	B (95% CI)			B (95% CI)		B (95% CI)		B (95% CI)	
Frequency of IE									
Low frequency (0–2 days)	0 (Ref)			0 (Ref)		0 (Ref)		0 (Ref)	
Moderate to high frequency (≥3 days)	0.46 (0.29, 0.63) ***			0.32 (0.14, 0.50) **		0.43 (0.26, 0.60) ***		0.33 (0.15, 0.51) ***	
Moderate frequency (3–4 days)	0.38 (0.16, 0.60) **			0.27 (0.05, 0.50) *		0.31 (0.09, 0.53) **		0.24 (0.01, 0.46) *	
High frequency (5–7 days)	0.54 (0.32, 0.76) ***			0.37 (0.14, 0.60) **		0.55 (0.34, 0.76) ***		0.42 (0.19, 0.64) ***	
P for trend	<0.001			<0.001		<0.001		<0.001	
Frequency of EFM									
Low frequency (0–2 days)	0 (Ref)			0 (Ref)		0 (Ref)		0 (Ref)	
Moderate to high frequency (≥3 days)	0.96 (0.66, 1.27) ***			0.79 (0.46, 1.11) ***		0.77 (0.47, 1.08) ***		0.59 (0.27, 0.91) ***	
Moderate frequency (3–4 days)	0.92 (0.53, 1.31) ***			0.75 (0.34, 1.15) ***		0.66 (0.28, 1.05) **		0.50 (0.10, 0.90) *	
High frequency (5–7 days)	1.03 (0.56, 1.50) ***			0.81 (0.32, 1.31) **		0.94 (0.47, 1.40) ***		0.68 (0.20, 1.17) **	
P for trend	<0.001			<0.001		<0.001		0.001	
	**Personal happiness (0–10)**		**ACC (0–10)**				**Psychological distress (0–12)**		
	**Model 1**	**Model 2**	**Model 1**		**Model 2**		**Model 1**		**Model 2**
	**B (95% CI)**	**B (95% CI)**	**B (95% CI)**		**B (95% CI)**		**B (95% CI)**		**B (95% CI)**
Frequency of IE									
Low frequency (0–2 days)	0 (Ref)	0 (Ref)	0 (Ref)		0 (Ref)		0 (Ref)		0 (Ref)
Moderate to high frequency (≥3 days)	0.29 (0.10, 0.48) **	0.22 (0.02, 0.42) *	0.41 (0.24, 0.58) ***		0.36 (0.18, 0.54) ***		−0.45 (−0.70, −0.19) **		−0.34 (−0.61, −0.07) *
Moderate frequency (3–4 days)	0.17 (−0.07, 0.41)	0.11 (−0.14, 0.36)	0.35 (0.14, 0.56) **		0.33 (0.11, 0.55) **		−0.30 (−0.62, 0.03)		−0.18 (−0.51, 0.16)
High frequency (5–7 days)	0.40 (0.16, 0.64) **	0.32 (0.07, 0.57) *	0.46 (0.25, 0.67) ***		0.40 (0.17, 0.62) ***		−0.58 (−0.90, −0.27) ***		−0.50 (−0.83, −0.16) **
P for trend	0.001	0.01	<0.001		<0.001		<0.001		0.003
Frequency of EFM									
Low frequency (0–2 days)	0 (Ref)	0 (Ref)	0 (Ref)		0 (Ref)		0 (Ref)		0 (Ref)
Moderate to high frequency (≥3 days)	0.54 (0.20, 0.88) **	0.42 (0.06, 0.77) *	0.48 (0.18, 0.77) **		0.28 (−0.04, 0.59)		−0.8 (−1.25, −0.34) **		−0.61 (−1.09, −0.13) *
Moderate frequency (3–4 days)	0.53 (0.10, 0.96) *	0.43 (−0.01, 0.87)	0.38 (−0.001, 0.76)		0.18 (−0.21, 0.57)		−1.02 (−1.59, −0.44) **		−0.86 (−1.45, −0.26) **
High frequency (5–7 days)	0.55 (0.04, 1.07) *	0.35 (−0.19, 0.89)	0.62 (0.17, 1.08) **		0.40 (−0.08, 0.88)		−0.48 (−1.17, 0.22)		−0.16 (−0.89, 0.57)
P for trend	0.003	0.05	0.001		0.08		0.005		0.09

FACC—family adversity coping capability; ACC—adversity coping capability; IE—individual exercise; EFM—exercising with family members; B—unstandardized regression coefficient; CI—confidence interval. ^1^ Missing data were excluded. Data were unweighted. The sample size for regression was 1911. ^2^ Model 1 was adjusted for sex, age, educational level, employment status, household monthly income, housing type, and number of cohabitants. ^3^ Model 2 was Model 1 with mutual adjustment for IE and EFM. *—P < 0.05; **—P < 0.01; ***—P < 0.001.

## Data Availability

The data presented in this study are available on request from the corresponding authors. The data are not publicly available because our analyses and paper writing on the results are in progress.

## Note

1 Given that responses for IE may include EFM, some measures were taken to minimize this issue during data collection. First, the surveyor emphasized that the question for IE referred specifically to the respondent themselves in both the landline and mobile surveys. In the online survey, the word ‘you’ in the question for IE and the words ‘with your family members’ in the question for EFM were bolded to emphasize that the question for IE referred to the respondent’s own situation.

## Abbreviations

The following abbreviations are used in this manuscript:

IE	individual exercise
EFM	exercising with family members
ACC	adversity coping capability
FACC	family adversity coping capability
PHQ-4	4-item Patient Health Questionnaire
FamCov-1	family amidst COVID-19 Survey
FamCov-2	family amid COVID-19 Survey 2
FHInTS	Hong Kong Family and Health Information Trend Survey
PA	physical activity
SD	standard deviation
MLR	multiple linear regression
CI	confidence interval
